# Extra-Limites

**Published:** 1824-03-01

**Authors:** 


### EXTRA LIMITES.

### I.

*Facts and Observations in Refutation of Sir Gilbert Blane's Doctrines as to the Contagious Nature of Yellow Fever.* By A. Musgrave, M. D. of Antigua; Physician to His Excellency the Governor, and formerly President of the Royal Medical Society of Edinburgh.

TO

THE EDITOR OF THE MEDICO-CHIRURGICAL REVIEW.

> "He studied well the point, and found
> "His Foe's conclusions were not sound,
> "From Premises erroneous brought,
> "And, therefore, the deduction's nought."—Swift.

Sir,

Your Number for March has just put me in possession of the only notice, which has yet reached this part of the world, of Sir Gilbert Blane's last Dissertation on Yellow Fever, wherein he so warmly renews the discussion of the contagious nature of that disease; and, as some weeks, at least, must necessarily elapse before I can obtain a perusal of the work containing it, I shall, at once, avail myself of the facility afforded by the "Extra Limites" department of your valuable Journal, to disseminate, more widely and expeditiously than could be effected through any other medium, some antidote to the poison which opinions, emanating from authority primâ facie so conclusive, are too well calculated to instil into the public mind—opinions (it must be admitted) not supported "with that dignified coolness and candour, which *becomes* a liberal profession."* I shall, nevertheless, endeavour, in this communication, to refute them, not by intemperate assertions or any train of reasoning which there is too much cause to presume would be again summarily dismissed as "shallow and perverted;"† but, by a plain exposition of facts of such authentic and incontestable character, as must render all reasoning superfluous.

---

* Blane's Medical Logick, 2d edition, p. 183.

† Medico-Chirurgical Review for March, 1823, p. 741.

In order to accomplish my object in the least exceptionable manner, I purpose (in the absence of Sir Gilbert's last publication) to examine, in succession, the validity of each of his strongest points, as set forth in the 2d edition of his Medical Logic, page 228, or "those considerations" which, he thinks, (even should every other view of the subject fail in proving decisive) "must, in minds prejudiced or unprejudiced, effectually cast the balance in favour of contagion"—and in doing this, I shall, I think, make it appear most convincingly to your readers, that, in medicine as well as in religion, the conduct of preceptors does not always supply the best practical illustration of the truth of their doctrines—for if it be conceded (as I apprehend it must) that no logic can be worse, or in other words, no reasoning more fallacious or unsound, than that by which positive and highly important conclusions are deduced from premises, not only doubtful, but absolutely false; then will it presently be shewn (as far as recent occurrences are allowed to operate in proof) that Sir Gilbert Blane, in his writings on the Contagious Nature of Yellow Fever, has most illogically betrayed a total disregard of those very precepts which he has himself been at such pains to inculcate.

"It is a melancholy truth, that there is, perhaps, no department of human knowledge in which there is so great a want of correctness with regard to recorded observations, as well as reasonings, as in medicine. We ought, therefore, to be strongly fenced against the inroads of error in others, as well as ourselves."—*Blane's Medical Logic, 2d edition, p.* 242.

But to proceed.—The first consideration which Sir Gilbert conceives to be decisive in establishing his favorite doctrine, he gives in the following words:—"The evidence in proof of its being actually imported from sea by particular ships, communicating it to the spot where the landing is made, and spreading from them to all communicating parts, whether contiguous or remote."

Could such evidence be in reality obtained, the question would undoubtedly be silenced at once and for ever; but I have no hesitation in affirming, that this confident but gratuitous assertion is directly at variance with the experience of every practitioner now resident in the West Indies.*

---

* This remark applies more particularly to the last nine years, the term of my own local knowledge and observation—and it may not, perhaps be deemed altogether irrelevant, or an unpardonable degree of egotism, if I take an opportunity in this place, with reference to Sir Gilbert's contemptuous allusion to my former communication, to state those circumstances which I regard as affording me some fair pretensions to credit from the profession at large. It is most certainly unfortunate that I can

In my letter to Dr. Fergusson, detailing the history of the epidemic which prevailed in Antigua in 1816-17, I stated, that no suspicion of the disease having been introduced by importation had been even hinted at by any individual whatever, and I, at the same time, adduced documents to establish that such could not, under the circumstances of its first appearance, have possibly been the case. I also adverted to the conclusive inference to be drawn from the perfect immunity enjoyed at that time by the Islands of St. Kitts, Nevis, and Montserrat, under a constant intercourse with us, unrestrained by any law or measure of precaution.

Subsequently to 1817, none but sporadic cases of the more concentrated forms of fever have been met with in St. John's, but our neighbours just alluded to have not been equally fortunate. In 1818, a most malignant and fatal epidemic raged at St. Kitts. The rate of mortality was exceedingly great—persons who had escaped the disease here in 1816, fell victims to it there—vessels arrived at this Port daily, with these melancholy accounts—nay, a fatal case was actually imported into the most sickly part of the Island, (English Harbour) and yet we saw nothing of those "tragical effects" which have given rise, on other occasions, to Sir Gilbert's "deepest grief and concern." In pursuance, however, of my plan, which forbids my advancing any thing unsupported by facts resting on irrefragable testimony, I shall, at once, refer your readers to an annexed extract from Deputy Inspector Hartle's Half-yearly Report for June, 1821, as principal medical officer in this Island, transmitted officially to our Governor, Sir Benjamin D'Urban, and by His Excellency's kind permission and Dr. Hartle's friendly sanction, at present in my possession, to be used in any manner that may be most con-

---

advance no claim to the character of a medical veteran—nay, so far from being able to do so, I am obliged to acknowledge that I must be at least twenty years longer a sojourner in the world before the whole sum of my existence can equal the already completed period of Sir Gilbert's "professional meditation and experience." I have however been *nine years*, as just stated, extensively engaged in tropical practice, and, during the whole of that time, independently of a wide range of private attendance, have had charge of the only hospital on the civil establishment of the Island, (into which all seamen from merchant vessels are received) and generally acted for the military stationed in St. John's.

In addition to these advantages, I reckon it not a little, that through the kind attention of our Governors, Major-Generals Ramsay and Sir B. D'Urban, I have had free access to all official documents relating to any epidemic diseases which may have chanced to prevail within the circle of their military command, and have thus been enabled to compare my own impressions and the results of personal observation, with the sentiments and experience of others.

ducive to the result I profess to have in view—The counteraction, namely, of "baneful impressions" which it is impossible for Sir Gilbert himself to deprecate more fervently or sincerely than I do. (*See Appendix* A.)

So forcibly does the extract here referred to, appear to me to bear upon the question—so deserving in every point of view to be considered an "experimentum crucis," that I cannot help enquiring, before I proceed with my proofs, whether, if Sir Gilbert himself had been called upon to devise some *single* test on which he would be satisfied to rest the whole merits of the case, he could possibly have dictated one more trying or unexceptionable? To my limited comprehension, it would seem peculiarly calculated to bring instant and full conviction to every candid and impartial mind, even if it stood alone and unsupported. That it fortunately, however, does not stand alone, the narratives comprised in the Appendix B. will abundantly testify.

To the truth and accuracy of every particular recorded in these documents, I most cordially subscribe. The facts, although not occurring under my immediate observation, were completely within my knowledge. They were matter of public notoriety, and the subject of general interest and conversation among all classes of the community. Captain Newcombe and his officers visited St. John's repeatedly during the prevalence of this fever among their crew, and repeatedly have I had the pleasure of meeting them in society, without observing that the slightest apprehension was excited lest the disease should be thus transported to this part of the Island; and the result most fully justified the implicit reliance of the inhabitants on its non-infectious nature.

During the convalescence of a midshipman attached to the Pyramus, I was consulted by a gentleman of large family, (most of its members recently from England) on the expediency of having the young gentleman removed to his house in a distant part of the country, as there existed some connexion between their families, and he was anxious to contribute to his rapid recovery by change of air. I frankly stated my firm conviction, that no danger would be incurred by such a measure, but, at the same time, requested that, if he himself entertained any doubts upon the subject, he would be guided by his own opinion. He, however, thought proper to act upon mine. The young man was accordingly, at once, removed from an hospital, crowded with patients in every stage of this malignant and fatal fever—highly malignant and fatal as will appear from the annexed returns*—where he had just

* Vide Appendix B.

suffered an attack in his own person; yet the gentleman's family into which he was received continued to enjoy uninterrupted health.

So far, I think, it has been satisfactorily shewn, that the disease cannot be imported into an island by any ship or vessel so as to infect the healthy inhabitants. The Appendix C, containing a letter from my friend Dr. Crichton, formerly principal Surgeon to the Naval Hospital at English Harbour, and at present engaged in private practice in this town, will sufficiently establish, that it is equally impossible, on the other hand, to communicate it from an island to the crew of any ship or vessel which carries with her no local cause in her holds—and this statement of Dr. Crichton's is amply confirmed by the singularly decisive fact (more irresistible than volumes of argument) to be found in Dr. Dyett's History of the Montserrat Epidemic, (*see Appendix* D.) where he mentions, that a young European, named Farrile, sickened with yellow fever the day after he embarked for England on board the merchant ship Arethusa, in August 1821, (the disease being then prevalent in the Island) and died, at sea, with every malignant symptom of the disease, the crew continuing, during the voyage, exempt from every thing like complaint.

Sir Gilbert's SECOND CONSIDERATION, decisive as to the contagious nature of yellow fever, is "The established fact, that its progress can be arrested by the separation of the infected from the sound"—to disprove the validity of which, even divested of its intimate connexion with the first, which has just passed under review, will require a very brief discussion.

It will be remembered, that the first cases which ushered in the epidemic of 1816-17 in this Island, were removed from St. John's, as soon as they were discovered, to the Hospital situated on Rat Island, a small insulated Rock considerably and directly to leeward of the town—that at the time of their removal, and, indeed, for several days afterwards, no other cases were heard of; and, when such did occur, it was decidedly established, by the most minute investigation, that the individuals affected had held no sort of communication with those who had been first attacked.*

It will further be remarked in the several narratives detailed in the Appendices to this communication, that the *sick* were repeatedly landed from the different vessels without the slightest effect in arresting the progress of the disease—while a removal of the *whole crew* never failed, at once, to put an end to its prevalence; notwithstanding the intercourse between the

* Medico-Chirurgical Transactions, Vol. IX.

sufferers and those who had escaped, continued altogether without restriction:—And, lastly, the separation which alone can be effectual—that from the local causes—will be found clearly pointed out in the circumstances stated by Dr. Dyett as occurring in the family which first suffered from the epidemic of Montserrat. After losing two of their number, the survivors of that family fled in alarm to the country, where they, and all around them, continued for some weeks in perfect health, but as soon as a deceitful pause in the succession of patients in town allured them back to their former residence, four fresh cases (one of them fatal) furnished a lamentable proof of the circumscribed source of this most dreadful malady.*

I could multiply, to any extent, examples of this kind, but even those already enumerated may probably be deemed superfluous; for it surely can require no subtlety of argument to demonstrate, that over the progress of a disease, which cannot be extended by the most unlimited intercourse, separation can exercise no influence, however trifling—and that yellow fever cannot be propagated by intercourse, I trust I have already established to the satisfaction of every rational mind.

I shall, therefore, dismiss this "consideration" by contrasting the marked effect of separation where we had to contend against a disease, really imported and decidedly infectious.

I was, in 1819, requested by one of my professional brethren, to look at a patient of his, labouring under, what he stated, at the time, to be, aggravated varicella—I, at once, recognized a tolerably severe case of small-pox, and, on a full consultation of the more experienced practitioners, it was unanimously decided to be so. We ascertained, beyond a doubt, that a member of the family, to which this person belonged, had recently arrived from St. Bartholomew's, where the disease was extensively and fatally prevailing, and where he himself had gone through an attack. Five of the number in the house which he inhabited had not been vaccinated, and they all subsequently took the infection. They were, however, successively removed, under the authority of the Commander-in-Chief, to Fort James, (which bears the same relative position as Rat Island, being directly to leeward of the town) and one case only occurred afterwards in their neighbourhood. It was under the confluent form. The subject was a girl, about fourteen years of age, and a patient of my own, whose friends peremptorily objected to her being taken

* See, also, my Note to Dr. Dyett's History of the Montserrat Epidemic, Appendix D.

from St. John's; but, by preventing all communication, except with the vaccinated or those who had gone through small-pox, an effectual stop was put to the dissemination of this most loathsome distemper; although, from the difficulties encountered in rendering the practice of vaccination general, I have little doubt that, at least, one third of the whole mass of our population would have been, at that time, obnoxious to its influence, had the disease been permitted to continue its progress undisturbed.

Sir Gilbert's THIRD CONSIDERATION, which next presents itself for examination, is "The difference of this pestilential form of the disease from the other two, in rendering those who have gone through it unsusceptible of a second attack.

To enter into a laboured refutation of this supposed peculiarity, would be decidedly out of place here, where recent facts, and such as are not yet before the public, are alone intended to be recorded.* The results of my own observation are already before the medical world; and Dr. Bancroft has, in my opinion, silenced every reasonable doubt on the question, in the Sequel to his Essay published in 1817. One additional and fatal proof, however, of the fallacy of such a notion, will be found among Dr. Hartle's Observations on the Fever which prevailed on Board the Pyramus Frigate.

Sir Gilbert's FOURTH CONSIDERATION appears to me so frivolous, as scarcely to merit serious refutation; for we find it gravely laid down as a chief means of distinguishing between diseases, contended to be essentially different, and to require opposite modes of treatment, that "greater numbers are affected by the one, and with symptoms more intense and mortal."

Now, I must here be permitted to use the language of the veteran Baronet himself, and call upon all persons of "common understanding, whose minds are not indelibly imbued with the dogmas of *contagion*," to say, whether this augmented intensity and general prevalence are to be considered as establishing such distinction, or whether it would not be more consonant to reason to infer from them, that the difference is only in degree, and that the more aggravated type supposed to

---

* I have, in conformity with this professed resolution to trouble your readers with nothing which has been already presented to them through any other medium, purposely avoided all reference to recent publications, more particularly those of Dr. Jackson and Mr. O'Halloran, on the late Fevers in Spain, which so amply corroborate the opinions I am here attempting to support. Dr. Jackson states, that during the epidemic of 1820, twenty well-authenticated instances came within his knowledge, of persons being attacked who had had the disease before.

constitute a disease sui generis is in fact nothing more than a highly concentrated form, which the ordinary remittent can assume under particular circumstances.

To say the truth, nothing can be more inconsistent or absurd than the different attempts at establishing diagnostic marks between these diseases to be found in the respective works of those who deny their identity. At this moment, while committing these hasty observations to paper, I have before me the various descriptions promulgated by the leading advocates of Sir Gilbert's doctrines, whom, as professing similar opinions, we should expect to agree, at least, in their exposition of those grounds on which alone such opinions could be legitimately founded. Yet, so far from observing this essential coincidence, we find them materially at variance, and meet at every page with endless contradictions. Dr. Chisholm tells us, that black vomit is improperly considered characteristic of the Bulam fever, and Sir Gilbert thinks with him. Mr. Pym declares that it is the grand test by which the nature of the disease is to be decided. Dr. Chisholm describes the discolouration of the skin in his malignant pestilential fever as being *dark* or *dingy*. We find the same thing compared, by Mr. Pym, to the tint of a *pale* lemon. Retention of urine is, by one, considered distinctive of the infectious form; but, by another, is enumerated among the symptoms of the bilious remittent. The seat of the headach—the state of the bowels—the varieties in the pulse—and extent of the hæmorrhages, are attempted to be called in aid by all, but their peculiarities are applied in the most opposite manner by each. I shall, therefore, in concluding the discussion of this 5th consideration, only entreat the juniors of the profession, destined for Tropical practice, and whom I can conceive to be wading in anxious uncertainty through the troubled sea of contradictory statements, to be assured, on the faith of an honest man, actuated by motives as pure, and a sense of duty as imperative, as any which can be supposed to influence the most "grateful servant of the State," that I have frequently, during the epidemic of 1816, and in the course of one round of visits, seen delineated in different cases (undeniably originating in the same pestilential cause, whatever that might be*) every shade of those varieties described by Drs. Chisholm, Pym, and others—these varieties or modifications being obviously and solely the offspring of

---

* I am warranted by Sir Gilbert himself in using the word *pestilential* to express only "a very high degree of prevalence, together with an extraordinary and calamitous rate of mortality."

diversities in the ages, temperaments, or assimilation of those affected. I can further declare, with equal confidence, that I have, since that time, met with cases, purely sporadic, which in their progress and termination have followed the course of the most malignant forms described as the true or infectious Bulam fever. One most remarkable instance of this kind occurred about two years ago. The subject was a carriage painter, named Higgins, about 30 years of age, and twelve months from England. After fatigue and exposure to the sun, he was attacked with fever, but unfortunately did not apply for assistance till the commencement of the third day. The case, even at my first visit, I pronounced to be lost. The skin was acquiring the dingy discolouration of Chisholm, and an unconquerable irritability of stomach indicated the approach of mortal symptoms. In the progress to dissolution, there supervened black vomit, hiccough, petechiæ, and profuse hæmorrhages from the nose, mouth, and intestines. The pains in the head and legs were exactly as pointed out by Chisholm, (the former being confined to the forehead and orbits, the latter to the calves) and the peculiar dilatation of the pupil, so much insisted on by that author as never accompanying the marsh remittent, was well marked—indeed, it would be difficult to conceive a more complete assemblage of every malignant indication; yet this man died in the quarters of a serjeant of the 5th regiment at the barrack immediately to windward of the town, surrounded by the wife and family of the latter, and visited by European soldiers and other friends. His bedding, &c. remained in use after ordinary cleansing, but we heard of no other case for months.

Whatever may be their discrepancies, however, in other points, the advocates for contagion would seem to be *unanimous* in admitting, that those fevers must be considered as possessed of that property which "prevail in a much more fatal degree than usual, sparing few who are within their influence, and carrying off a large proportion of the whole population."*

Now if these be, indeed, the true and grand characteristics of that "destructive monster" described by Chisholm and others, it will not, I presume, be denied, that the fevers alluded to in this communication were of a similar type; for they were, God knows! general enough among those exposed to their influence, and nearly *one*, on an average, out of *three* who were attacked, fell a victim to the extreme malignancy of the symtoms.† But I really find some difficulty in per-

* Blane's Medical Logick.

† See Returns annexed to Appendix B.

suading myself to argue this point dispassionately, for is it not insufferable that we, who live in the very hot-bed of the disease—who witness its ravages and are ourselves exposed to its influence—are to be dogmatically told what is or is not yellow fever, by men (however respectable or eminent they may be) who deal out their doctrines from the comfortable closet and salubrious atmosphere of an English residence? Yet, as by patience alone, we can hope for eventual success in eradicating those "baleful impressions" which the plausible, but hollow arguments of the contagionists have unhappily made on the minds of those, who, on their arrival in the West Indies, view with horror the approach of the simplest fevers—and as it has hitherto been my study in this communication to postpone, on every question, "contentious argumentation"* to the relation of facts, I have appended extracts from official documents, to prove that the Tobago fever, specified by Sir Gilbert as exemplifying by its "tragical effects" the necessity for quarantine regulations,† was considered, by those best qualified to decide upon its real nature, as proceeding exclusively from the concentrated influence of marsh effluvia, and to be altogether destitute of contagious properties. And, as it respects the Island of Barbadoes, where so "consolatory an exemption" is said to have been enjoyed, I can only declare that, as far as I have been able to learn, this exemption proceeded simply from the inactivity, at that particular time, of the local causes, as happened with us, while our neighbours, St. Kitts, Nevis, and Montserrat, were suffering so severely;—and that the only happy effect I could ascertain to have resulted from the "separation" there enforced was, that an unlucky field officer, who had lost one of his daughters, was made to enhance his misfortunes by the destruction of his bedding, and the greater part, if not the whole of his furniture, when these articles, had they been exposed to public sale, would not have been considered by the inhabitants as deteriorated one shilling in their value, by the death which had excited such excessive but groundless alarm. *(See Appendix* E.)

I have now arrived at the 5th and last of those Considerations, supposed by Sir Gilbert "effectually to cast the balance in favor of contagion"—namely—"that there are well attested examples of its (the yellow fever) being communicated from one ship to another in the middle of the ocean."

---

* Blane.

† Blane's Medical Logick, 2d edition, p. 235.—"In the Island of Tobago, there died, last year, of *this epidemic*, more than two thirds of the troops which had arrived from England," &c.

That many examples, *at first view* admitting of such a construction, may be adduced, I am ready to concede; but, that even *one* can be found which will, *after minute investigation*, bear upon the point at issue, I must be permitted to entertain very serious doubts. The most superficial view of the subject will suffice to shew that, to these ship fevers principally, is to be traced the groundwork of those endless controversies which have, from time to time, been agitated on the contagious nature of this fever—while, at the same time, a moment's deliberate reflection ought to demonstrate how fallacious must be the conclusions drawn from a source so encompassed on every side, by circumstances calculated to mislead.

It will not surely be contended that, in order to prove a disease to be infectious, it would be sufficient merely to establish, that, after it had been accidentally introduced into a particular place, cases of the same description were observed to occur; when, it is equally undeniable, that a like prevalence of a similar disease might have, or indeed had, on other occasions, happened unconnected with such an introduction.* To confirm, therefore, the claim of any one of Sir Gilbert's facts to be classed as an "experimentum crucis," it would be essential to prove the impossibility of those vessels, to which he has alluded, having carried within themselves that deleterious accumulation which it is now acknowledged on ample evidence can exist in the holds of vessels, to the destruction of the majority of their crew, with fevers of the most aggravated kind.

The fact is, that the contagionists, and not their opponents, have erred, "from confining their views to one spot, and not considering the question on a comprehensive scale in all its

---

* My highly respected friend Dr. Fergusson, very forcibly portrays the dangers which may too possibly result from disregarding this source of fallacy, in alluding to the Regalia transport, which arrived from the Coast of Africa at Barbadoes, in a sickly state, about a twelvemonth before the eruption of that epidemic which carried such devastation through most of these Islands in 1816. "I cannot pretend," he says, "altogether to assert that I have proved these points; for how few amongst the ever-changing phenomena of diseases admit of being subjected to the rules of evidence! And I am aware of how much I have been favored by circumstances, and of what a different interpretation the facts I have collected would have borne, had the present epidemic, which now afflicts the Islands, broke out, in the ordinary course of the seasons, a year earlier, at the time the Regalia was here: for these, instead of assisting me to elicit the truth in the manner they have done, would, in that case, have been turned to the confirmation of error and the perpetuation of the delusion in regard to imported contagion."—*Med.-Chir. Transactions, Vol.* VIII.

extent and bearings"*—for the stanchest advocate of non-contagion might have had his creed most seriously shaken, had he confined his views to the circumstances of those fevers, which I have adduced in evidence in this communication, previously to the several crews being removed from their respective vessels. While they continued to be exposed to the influence of local causes—the general prevalence of the disease—its progressive fatality—the impossibility of arresting it—every thing might have conspired to puzzle or mislead the most attentive observer—but it was the landing those people and their introduction into an island where no such disease existed at the time, which constituted the truly unexceptionable test of its non-infectious nature—the genuine "experimentum crucis"—and how repeatedly this test has afforded a result the most triumphant for the non-contagionists, has been shewn by the documents already referred to. Thus far I have argued assuming the accuracy of Sir Gilbert's statements on this point to be unquestionable. That it is far from deserving to be so considered, however, the examination instituted by Dr. Bancroft into the circumstances attending the fever on board the Hussar sufficiently proves.† We have, consequently, only doubtful testimony on the one side, while, on the other, the facts already alluded to on the authority of Drs. Crichton and Dyett, as occurring on board the Montagu packet in 1816, and the merchant ship Arethusa, from Montserrat, in 1821, (both of which vessels received on board, with impunity, fatal cases of yellow fever) will, I presume, be deemed evidence as conclusive as could possibly be demanded even by the most sceptical of that class of which our author may be considered the leader.

Sir Gilbert Blane's five "considerations" have now passed under review, and each of them, after the most attentive examination, has been shewn, not by *reasoning*, but by *facts*, to be destitute of foundation—so utterly destitute, indeed, that I can hardly believe it possible that any one of your impartial readers, after a careful perusal of the documentary evidence I have adduced, will deny my right to advance the following very opposite conclusions as incontrovertibly established.

1st. That yellow fever, or in other words, fevers bearing in every stage—in their commencement—progress—and termination—the characteristic marks of the most malignant pestilential form, described by Chisholm and others, "prevailing in a more fatal degree than usual, sparing few who are within

---

* Blane's Medical Logick.

† Bancroft on Yellow Fever, p. 769.

their influence, and carrying off a large proportion of the whole population,* "may, with absolute impunity be introduced into any island, place, or vessel, which, but for such introduction, would have continued healthy; and that, consequently, such fevers cannot possibly be contagious.

2ndly. That separation, except from those local causes which constitute the invariable source of yellow fever, can have no influence whatever in arresting the progress of the disease.

3rdly. That one attack of yellow fever by no means exempts an individual from all risk of suffering from it a second time; although the general liability to its influence is unquestionably in an inverse ratio to the period of assimilation.

4thly. That the more general prevalence of tropical fevers at one time, with symptoms more intense and mortal than at another, can only be considered an additional proof of their being all of them essentially the same, although they may at different times rage with varied degrees of violence and mortality from causes which it must be admitted have not yet been properly defined.

5thly. That as it has been shewn to be impossible to infect a healthy vessel by the introduction of yellow fever from the shore, so must it be equally impracticable to communicate it from one ship to another in the middle of the ocean.

It only now remains for me to take a cursory glance at the treatment of yellow fever, rendered necessary in my opinion by the solemn, but loose and dangerous language, so pathetically addressed by Sir Gilbert to the juniors in the medical service of the navy on the subject of blood-letting and purging.† A reference to the 9th volume of the Medico-Chirurgical Transactions will place your readers in possession of a more detailed account of my own ideas on the best practical measures to be adopted than there can be any necessity for my recording in this place. It will there, I think, appear to their satisfaction, that I am no advocate for "outrageous and indiscriminate practices," but, that both reason and experience have combined to render me fully aware of the mischief which may be done by the "rash and undistinguishing members of our profession"—yet I must at the same time declare it to be my settled opinion, that in the earlier stages of yellow fever, the safety of our patients is much more likely to be compromised by timidity than by decision—call it rashness, if Sir Gilbert so wills it—and that no precept more fraught with

---

* Blane. † Medico Chir. Review, for March, 1823.

danger can be impressed on the mind of an inexperienced practitioner, than that a "single bleeding," however copious, will suffice in the treatment of the severer forms of the disease. The case he details, I agree with him in pronouncing an instance of bad practice, but of bad practice originating not in the rashness he condemns, but in that very timidity his observations must inevitably inculcate; for had the two first bleedings been more vigorously carried into execution—the orifice large—the quantity drawn limited solely by its decided effects upon the system, and the second had succeeded to the first within three or four hours, or as soon as the vascular excitement had been re-established with a recurrence of headache, &c. I think it more than probable, that the life of this young commander would have been saved: had the severer symptoms however re-appeared, notwithstanding the second bleeding (admitting that his frame was not athletic as stated by Sir Gilbert) the lancet ought then, in my opinion, to have been thrown aside, and the case altogether confided to mercury, which, after such liberal depletion, would have afforded a very favorable prospect of success. But had his frame been robust, and his habit plethoric, I most certainly should not have hesitated to repeat the bleeding, *within the first eight and forty hours*, as often as the violence of the symptoms appeared to call for reduction. To sum up, blood-letting is, as I said on a former occasion, our sheet anchor in all Tropical fevers, affecting Europeans. But in proportion to their violence, or degree of concentration, must we be prompt and decided in its adoption and repetition, *at the very onset*. The remittent forms may be judiciously combated by venesection, as late as the third, or under particular circumstances, even the fourth day, but the concentrated, continued, or what is universally termed yellow fever in its worst shape, may be found in the rapidity of its progress to afford a period, not exceeding a few hours, which if permitted to pass over through indecision, this Herculean remedy would be afterwards rendered inadmissible. Mercury becomes then our only, but, alas! a most uncertain resource, and recent experience in the more aggravated remittents has convinced me, that this medicine, too, can only be depended upon in cases of this kind, when no limits are observed in its administration, but such as are indicated by an amelioration of the symptoms.

In concluding, I must be permitted to observe, that in annexing these facts and observations to the pages of your Journal, I have been actuated by no presumptuous desire to court notoriety, by obtruding myself into the field of controversy with a physician, so generally and deservedly respected as Sir Gilbert Blane. I profess myself to have been influenced simply by a regard to those sacred duties, which every conscientious man assumes when he embarks in the practice of medicine—and, could I have circulated the same information through the medium of some abler pen, I should most willingly have avoided thus presenting myself before the public. My letter to Dr. Fergusson was written without the slightest idea that it would appear under the form of a distinct communication. It was merely intended to convey to a valued friend, a statement of facts to be used by him in support of doctrines which had my fullest assent, and were advocated by himself—and that friend, aware that unlimited confidence in his judgment would preclude my seriously objecting to any plan he thought advisable, had it read before the Medico-Chirurgical Society without consulting me.

A. MUSGRAVE, M.D.

St. John's, Antigua,
20th September, 1823.

### APPENDIX A.

In the year 1818, when yellow fever prevailed generally, and with great mortality, at St. Kitts, no impediment or quarantine restrictions, to the intercourse between this and that Island, were resorted to, nor do I believe thought of. One of the army vessels, removing from St. Kitts some military, received on board an officer of the 1st West India regiment, who, to all appearance, was in good health, but soon after the vessel sailed, this officer was taken ill with fever, and was landed here at 7 o'clock at night, (forty-eight hours after he was attacked) with every malignant symptom of approaching dissolution; he died the following day. In this army vessel there were many other officers, and two or three more embarked here, for Dominica; no precautionary measures were taken in this Hospital against contagion—the white or European orderly men were employed about him generally—hospital-assistant Ewing, who had only been fourteen months in the West Indies, visited with me, yet none of those who were on board the vessel or attended him in hospital had the fever. His effects were sold, and used by those who purchased them without dread of yellow fever.

### APPENDIX B.

The three following narratives are all derived from the same valuable source as the preceding—the Official Reports, namely, of my friend Dr. Hartle, recently promoted to the rank of Deputy Inspector, than whom a more zealous, humane, and indefatigable medical officer is not attached to His Majesty's Service, and whose long and successful experience within the Tropics, entitles every thing coming from him to the most attentive consideration. The three highly malignant and destructive fevers they embrace, I have included under one head, because, although occurring at different periods and under modified circumstances, they are all mutually and obviously illustrative of the facts and conclusions to be deduced from the origin and progress of each, when separately considered.

### FEVER ON BOARD THE DASHER, 1821.

" On the 26th of August last, His Majesty's armed transport Dasher left Carlisle Bay, Barbadoes, bound for Tobago, but, in consequence of severe gales of wind, she was obliged to go to St. Lucia. From thence she proceeded to Tobago, and received on board a company of the 9th regiment, and conveyed them to Grenada, that they might avoid the endemic fever of the former place. This company, while on board, was perfectly healthy. On their landing at Grenada, I have a public document now before me which proves that they arrived equally so—they were immediately placed in quarantine, and remained so for the space of fourteen days, during which period not a man was sick. The Dasher, after landing this company at Grenada, proceeded hither, but a few days before she reached this port, yellow fever made its appearance amongst her crew, and previous to her arrival, six men had been attacked, two of whom had died. In the evening of the 22d September, she arrived in English Harbour, and on the 23d, the assistant-surgeon, Mr. Maclean, reported two men ill with yellow fever. I directed them to be immediately sent to this hospital. On their arrival, I was not a little surprised to find both in the last stage of the disease, and labouring under all the *most malignant* and distressing symptoms. One died in a few hours. On the following day (the 24th) seven were attacked, through the day, all suddenly and seriously. This, and the nature of the disease, brought forcibly to my mind what the Childers suffered, from the putrid state of her hold. I immediately addressed his Excellency Major-General Sir B. D'Urban, reporting the circumstance, and recommending that, without loss of time, the officers, crew and marines, be ordered out of the ship and encamped on an isthmus of land at the mouth of the harbour, and to leeward of all the bay; where every communication could, with great ease, be suspended; and that black people be employed to take out all her stores, tanks, ballast, &c. &c. and clean the ship generally and thoroughly under my daily inspection. His Excellency immediately acquiesced in my proposal, and accordingly, as soon as the ship could be warped into the harbour, properly moored, and safely arranged, to permit the removal of her ballast, the officers, crew, and marines were landed, which took place on the 26th September. Two cases of fever occurred on that day. two on the 27th, one on the 29th, (which was the assistant-surgeon) and the last on the 4th of October, making in the whole *fifteen* cases, *four* of which died. Black people immediately commenced clearing out the ship; after removing the stores, tanks, furl, ballast, &c. the dirt was scraped together, but this amounted to no great quantity. This did not satisfy my mind, and I insisted on having the limber boards taken up. Some objection was at first made, as to the likelihood of their being broken, but I was determined to leave nothing undone, and they were accordingly removed. Here, to the astonishment of every one, lay the mischief. On taking up these boards, the noxious effluvia surpassed any thing that I had before experienced, and it was with difficulty that the blacks, who were accustomed to this work, could remain. The ship carpenter, who had been constantly accustomed to work in the dock yard, and on many such occasions, assured me, that he had not before experienced so putrid a smell from any ship's hold. Between the timbers there was a collection of carpenters shavings, &c. in great quantities. These had so completely choked up the limber holes that the water could not pass to the well of the pumps, and lay stagnant. The vegetable matter was, therefore, in a state of decomposition, and this, acted on by high atmospheric temperature, became neither more nor less than a marsh in the centre of the ship. The fever was, consequently, of similar type, (but more aggravated by locality) with that which we may expect from paludal effluvium. It may be worthy of remark that, on the following evening, I was called to see one of the blacks who had been assisting in cleansing out this putrid matter which lay under the limber boards. I requested Hospital-assistant Hall to accompany me. The man was evidently labouring under a similar type of fever with that of the Dasher's people, and narrowly escaped by my attention. When the ship had been thoroughly cleansed, washed, white-washed, and fumigated, the officers, crew, and marines were allowed to return to her.

"While they were encamped they continued free of disease, but soon after returning, the painting commenced, and Lieutenant Wolridge was obliged to leave the ship and sleep in the dock yard, and the first, or chief mate being in hospital, the crew soon found the way to procure new rum, and I have been credibly informed, that many of them have been seen at night drunk, lying on the water-side unable to move. What was the consequence? Not fever of the ardent type; but common continued, gastritis, dysentery, cholera, &c. &c. That the fever was generated on board by noxious effluvia received into crowded and badly ventilated berths, is, I think fully proved, for the moment the crew and marines were removed from the sphere of this hidden enemy, the disease ceased. Let it be remembered that the crew removed from the ship on the evening of the *26th September*, and, by examining the nominal Return, it will shew that *the removal, at once, checked its progress.* Nothing like the most distant appearance of contagion could be traced. Those who were brought to hospital were attended by myself and Hospital-assistant Hall, who had newly arrived in this country. One of the servants of the hospital was an European soldier of the 35th regiment, who constantly assisted the fever patients, dressed their blisters, gave injections, and attended to all their other wants, yet none but those residing on board the ship, or exposed to the effluvium from her holds, prior to her expurgation, suffered by the fever."

FEVER ON BOARD H. M. FRIGATE PYRAMUS, 1821, 1822.

"His Majesty's ship Pyramus left this port, as I understand, perfectly healthy, on the 19th October, in company with the flag-ship of Rear-Admiral Fahri, for St. Kitt's, where she remained until the 28th October, when she sailed from thence for this Island. A day or two prior to her arrival here, fever of a most alarming type made its appearance amongst the officers and crew. She came into English Harbour early in the morning of 1st November, and as soon as she was anchored, Captain Newcombe sent a lieutenant to inform me of the state of the ship, and to request medical assistance. I immediately went on board, and was surprised to find that an officer (lieutenant) had died the day before with only a few hours illness—that the purser and six men lay dangerously ill; the surgeon who, from his own account, had been attacked himself, was unable to perform any duty, and was then in a precarious state from extensive, and what appeared to me to be, depletion, without regard to its consequences; the others, therefore, had received no medical assistance beyond that of an ignorant surgery man, or what is called on board a loblolly boy. He had bled them, and given some cathartic medicine, but it did not appear to me that the bleeding had been either to a sufficient extent or from a proper orifice, and unfortunately, the time for its repetition was past, as the disease was in its secondary stage. To this, therefore, I attribute the misfortune of losing the three first attacked, (purser and two of the men) for organic derangement had already taken place. I immediately proposed the removal of all the sick, which Captain Newcombe instantly sanctioned, and as soon as the oppressive heat of the day had passed, they were landed and conveyed to the hospital. The two men who died suffered under all the *characteristic symptoms of yellow fever;* black vomit, hæmorrhage from nose, mouth, and anus. The purser had nausea, but no vomiting; he bled profusely from the nose, and, a little before he died, passed, involuntarily, a large quantity of black fetid blood, per anum. I lost no time in reporting the distressed state of the ship to His Excellency Major-General Sir Benjamin D'Urban, commanding His Majesty's Troops, and, at the same time, recommended, that one of the hospital-assistants doing duty in this garrison, should be ordered to take medical care of the ship. His Excellency was pleased to signify his approbation, and Mr. Freer was ordered on board. On the following morning, (2d November) I again visited the ship, and found that the lieutenant of marines had been attacked with fever in the night. He had been freely bled, and was then under the influence of cathartic medicine; I wished him to go to the hospital, but he solicited to be allowed to proceed in the ship, and fearing that a removal might distract his mind, I did not insist on his going on shore. He had so far recovered as to be convalescent when the ship arrived at Barbadoes. The Pyramus proceeded in the afternoon of the 2d November to Barbadoes. Mr. Freer, who returned to this garrison on the 2d instant, having been relieved by a naval surgeon, reports that many fresh cases occurred in the passage up, but that he had landed most of them at Barbadoes in a convalescent state; that when he left the ship the fever still prevailed."

The foregoing extract is made from Dr. Hartle's Official Report, dated December, 1821. In his next, for June, 1822, he thus resumes this interesting narrative.

" The dire effects of concentrated miasma were very recently and decidedly proved on board His Majesty's frigate Pyramus, which came into English Harbour in the month of November 1821, on her way from Saint Kitts to Barbadoes, to obtain medical assistance, and land some officers and men labouring under yellow fever. The disease had suddenly appeared, and I could not, at that time, discover any manifest cause for it. Neither officers or crew had been exposed to solar influence, or any other known predisposing cause. I was therefore fully convinced, in my own mind, that the source was in her holds, and in my report for December 1821, I suggested the necessity of cleansing them, and expressed my hope that it would be my lot to investigate this most interesting subject of research. On her arrival in Carlisle Bay, Barbadoes, the disease became so general and alarming, that Mr. Tegart, the inspector of hospitals, ordered a board of medical officers—' to examine into the state of that ship, and investigate the probable cause of the sickness that prevails on board. The board will also report upon what may appear to them the most likely means of checking the progress of the disease, or removing the cause of it.' In the first part of their report, the Board appear to attribute the cause, in some measure, to the coal-tar with which she was injected, for they particularly observed the offensive effluvium ' arising from that substance mixing in the hold with the bilge water.' In the second paragraph they remark, that the ship lay for thirty-four days, at different times, in English Harbour; and, in the third paragraph, consider, ' with respect to the predisposing cause of the disease, at present prevailing in the vessel, the Board are inclined to attribute something to her stay in English Harbour.' If the coal-tar, when mixed with the bilge water, produced so offensive an effluvium, it is clear to me, that it ought to have been considered as inimical to health;* and, with all due deference, I think, the Board would have acted more to the purport of their instructions, had they recommended an immediate expurgation, instead of advising that the ship should be taken to sea, ' and cruise in the vicinities of the islands:' for it does not require much ratiocination to prove, that until the cause is removed, the effect cannot (nor did it in this instance) cease. I would ask if thirty-four days stay in English Harbour, at different periods, caused this malignant disease in the Pyramus, why did the other ships of the squadron escape it; for they all lay a much longer time in the same bay, and the Salisbury, bearing the admiral's flag, remained for three weeks together, yet none of them were sickly? It may be here deserving of notice, that the only ships then on the station, injected with coal-tar, were the Pyramus frigate, Esk sloop of war, and Dasher transport; all of which suffered (the former and latter most severely) with a similar type of disease—*yellow fever, in its most malignant form.* Fortunately for the officers and crew of the Esk, soon after her arrival on this station, she struck on a sunken rock, on the Spanish coast, and so materially damaged her keel, that on coming into English Harbour every article was obliged to be taken out of her, to enable the

* *Note by Dr. Musgrave.*—It appears to me, that the coal-tar contributed to the production of this fever only in so far as it obstructed the free current of the water to the pumps. But this, I should imagine, would be deemed amply sufficient to preclude its being used in future.

shipwrights to heave her down, and get at the injury. Without considering the consequences, her crew were employed not only in this laborious work, but in that of cleansing her holds, which contained a quantity of stagnant bilge water, and offensive coal-tar; the latter obstructing the passage of the former to the pump-wells;—during the process of expurgation, yellow fever appeared amongst the people, and but few escaped an attack. As the Board of medical officers, at Barbadoes, had suggested the propriety of Captain Newcombe's taking the Pyramus to sea, he left Carlisle Bay, and cruised as far as 28 North; but, finding this avail nothing, and that the disease became more alarming, he hastened to English Harbour, and arrived here on the 3d of January, 1822. I anxiously embraced the opportunity to ascertain if my prediction was correct, and immediately visited the ship. On finding that the sickness continued, with increased violence, I requested permission to examine the vessel, which I did in the most minute manner; and it is but truth when I say, that no ship could possibly appear cleaner or in better order; and had I not been convinced that the cause was in her holds, I should have been at a loss, from the good order and apparent comfort of the people, to account for the cause of so malignant a fever. I instantly removed all the sick to the hospital, and wrote to Captain Newcombe, recommending the removal, as soon as possible, of all the officers and crew from the ship, and that black people should be employed to do the laborious work of removing her stores, shot, tanks, ballast, &c. &c. and cleanse (under my frequent inspection) the holds, and every part of the vessel, thoroughly;—Captain Newcombe acquiesced in my proposal, but as it required some days to dismantle, and place the frigate in a proper state of safety, they did not land until the 15th. This unavoidable delay exposed the people to the influence of the local causes much longer than I could have wished. His Excellency Major General Sir Benjamin D'Urban, with his usual benevolence and zeal for every branch of His Majesty's service, offered, as the Fort Berkly isthmus was not extensive enough for their encampment, to evacuate Middle Ground barracks for their accommodation; but Captain Newcombe foreseeing the impossibility of keeping his people together on this post, preferred to quarter them in the Eastern dock-yard, from whence they could not go, but by water. The seamen and marines were therefore quartered in the upper part of the Capstern House, while the lower part served as a store for such articles of the ship as could not be exposed to the weather. Unfortunately the ship lay alongside of this wharf. Anxious to ascertain, by ocular demonstration, the cause of this endemic, I daily visited the ship, and was present at the opening of the holds—the effluvia from which surpassed any thing I had ever before experienced. Zeal on the part of the first and second lieutenants, master, and surgeon, incited them to accompany me, and they were accordingly with me when the limber boards were taken up. It is not in my power to describe the stench which issued forth on their being raised. The boatswain, curious to see what we were doing, did not come down, but lay on the lower deck to view us. This confined gas rushed up the hatchway, where his head was hanging over, so offensively oppressive, that he fainted. In the night he was attacked by the fever, and narrowly escaped. On the following day the first lieutenant was attacked. In the evening the second lieutenant was taken ill, and, in a few hours after, the master and surgeon. The second lieutenant, I am sorry to say, fell a victim to the disease, on the fourth day. On the third day I was myself very much indisposed, but fortunately a spontaneous diarrhœa removed all my febrile sensations. Here we see every individual present at the opening of the holds and limber boards attacked by the prevailing disease; and if we had no other proof of these morbific miasmata being the cause, the instances adduced would, in my opinion, be sufficient to substantiate that they were so, and save us the trouble of searching for any other, or ascribing the most trifling agency to that bugbear contagion. From the 15th day, on which the officers and ship's company were landed, to the 30th January, only eighteen cases occurred; and I was vain enough to think I had succeeded in checking its progress, but, on the 31st of January, six fresh cases were received into hospital, and the disease again appeared with increased violence and malignancy. This sudden re-appearance and violence of the disease induced me to believe that the people had some secret communication with the ship, which was then undergoing a general expurgation. This, with a little trouble and exertion, I ascertained to be the case, and instantly recommended to Captain Newcombe to occupy the upper part of the Western Capstern House for the convalescents, and encamp the remainder of his people on the Fort Berkly isthmus. Accordingly, on the 6th February (as soon as the encampment could be formed) they removed to the camp; this change of quarter and situation had the desired effect, as will be seen by my subjoined extract. Although I was fully persuaded, in my own mind, that the cause of the disease lay in the ship's bottom, I had not an idea, that such extensive bogs could have been found under the limber boards, and between the timbers. It would appear scarcely credible, that four large mud boats of filth should be taken out of this frigate, which had only been six months from England, and, I believe, not long out of dock. This, however, is easily explained. The shipwrights had never taken out their chips and shavings—the coal-tar, from heat of climate, had run into all the limber-holes, and so cemented those chips and shavings, that no passage remained for the water to go to the well of the pumps; it, therefore, lay stagnant under a heat of one hundred degrees of Fahrenheit's thermometer. As soon as the limber-boards were raised, I requested that the pumps should be worked. Both chain and hand-pumps were used, and when they could no longer draw, the water and mud-like matter remained nine inches deep, just the depth of the timbers in the ship's bottom. Each of the holds presented similar matter, but the after magazine, which lay immediately under the gun-room, surpassed all; it appeared as if this confined place had been intentionally stuffed with chips and shavings, all of which lay in stagnant bilge water, mixed with coal-tar. The stench from it was so oppressive that, with difficulty the blacks could be kept at work to remove it, and they were frequently obliged to run on the quarter-deck for pure air. This readily accounts for the fact that every officer's servant, and servant of the gun-rum mess, suffered with the fever, and most of them died. During the process of expurgation, three of the blacks were attacked by fever, evidently characteristic of that which prevailed amongst the ship's company. One, particularly, who was employed in cleaning out the dirt, coal-tar, and stagnant water, which lay in the after magazine, suffered most severely. The irritability of stomach exceeded any thing of the kind I had before witnessed in one of his colour, and it was by the greatest care and attention that he recovered."

Dr. Hartle proceeds to state that, after the ship had undergone a thorough expurgation, the officers, seamen, and marines re-embarked on the 11th of March, in health and spirits, and had continued so up to the date of his Report. I shall, therefore, pass on to the next narrative.

FEVER ON BOARD A TRANSPORT, FEBRUARY 1822.

" In the month of February, some recruits for the European regiments serving in this command, arrived at Barbadoes, and in March following, the ship arrived here with those for the 35th regiment, and 9th foot. On the passage from Barbadoes yellow fever made its appearance amongst the troops, and, on the ship's coming to anchor in English Harbour, seven cases, in the second stage of the disease, (who died with black vomit, hæmorrhages, and petechiæ,) were sent on shore to this hospital. Indisposition prevented my visiting the ship on that day, but Mr. Munro, surgeon of the 35th regiment, did; I desired that he would send every individual he found complaining to hospital—accordingly two were sent on shore. Prior to Mr. Munro's inspection, Dr. Barclay, who came from Europe in medical charge of the recruits, wrote to me reporting that he had an officer (Major Loftus, of the 9th regiment) dangerously ill on board. I desired that he should be immediately landed and brought to hospital. On his arrival, I was surprised to find that a very short period would terminate his life. On the morning of the 11th I visited the ship myself, I found her very clean, and in good order; two more men had been attacked, whom I brought to hospital, and instantly wrote to His Excellency Major Gen. Sir B. D'Urban, commanding His Majesty's troops, recommending that, as the transport was to go to Saint Kitts prior to proceeding to Grenada, the detachment of the 9th foot should be landed and encamped on the isthmus of Fort Berkly, until the ship returned, as the disease appeared then to be confined to the 9th only; but, in the evening, six fresh cases were landed, belonging to the 35th regiment and Royal Artillery. This induced me again to address His Excellency, recommending that the detachment generally should be landed and encamped, which His Excellency was pleased to sanction, and in the morning of the 12th they disembarked, and took up the encampment. The ship underwent a partial cleansing, and proceeded to Saint Kitts; after landing the stores and baggage, she returned to English Harbour on the 19th of March, and had a general purification. The detachment for Grenada re-embarked on the 23d, and sailed immediately. It arrived in good health, and, I understand, has continued so. The staff surgeon, by my advice, placed them, on their arrival, under observation for some time. On the 24th of March, the detachment belonging to the 35th regiment marched from the encampment to the ridge, and joined the head quarters of their regiment. Not a case of ardent fever has occurred amongst them.

It is a pleasing reflection, and a source of great gratification to me, that notwithstanding *one hundred and forty-seven cases of yellow fever, as distressing and malignant as any I have before witnessed*, have been by these vessels imported into this island since September 1821; we have not heard of a single instance of any individual, but those directly exposed to the local causes, having been attacked. At the time the Dasher's sick were received into this asylum, (September 1821,) European orderlies attended them, and Mr. Thomas Hall, my zealous and indefatigable assistant, had only been a few months from Europe. With all these patients we never apprehended danger—we took no precautionary measures—it was our duty to exert every effort for the saving of lives, valuable to the service—we were day and night in the wards—we not only administered the medicines, but we assisted the nurses—we gave injections; we applied and dressed blisters; we assisted in putting the patients into, and out of warm baths; we sat on the bed-sides of the sick, to cheer up their spirits; we have been frequently vomited on, by those labouring under black vomit; we examined every case that died:—yet, all this exposure never produced the smallest indisposition beyond that resulting from fatigue and want of sleep. In the foregoing pages I have shewn strong and sufficient proof that the yellow fever, which prevailed in His Majesty frigate Pyramus, was generated in her holds—that the boggy matter there accumulated emitted effluvia sufficiently deleterious and concentrated to affect the boatswain, who suffered instantly and severely by being casually exposed to it, when disengaged from the limber holes—that each individual present at the opening of those holds and limber boards were attacked, and that the blacks who were employed to cleanse the ship (people not at all subject to fever) were assailed by the same disease. I have also substantiated the advantages resulting from a removal; and separation of the crew from the local causes—for my abstract will show that until this change took place the disease could not be checked. If, therefore, this disease had been contagious, a removal, particularly to so short a distance, (two or three hundred yards,) could not have arrested its progress. Besides this, the gentlemen of the island visited the officers—the officers returned their visits. The people of the country formed a vegetable and fruit market in the camp. The hospital bedding was not destroyed—it was, as is done in every other instance of sickness, washed and aired. It has been in constant use ever since by both the navy and army. Yet this fever has not appeared in any individual, but those directly exposed to the original cause. Since the expurgation, the ship has continued remarkably healthy. On the 14th November a sporadic case of ardent fever, occurred on board—it proved *fatal*, with *every distressing symptom characteristic of yellow fever;* yet the same individual had suffered most severely in the month of March, when the endemic prevailed so generally in the frigate."

ABSTRACT of the daily Admissions of Yellow Fever Cases, into the Army General Detachment Hospital, from His Majesty's Ship PYRAMUS, in English Harbour, ANTIGUA.

| 1822. | 3d January | 5th January. | 6th January. | 8th January. | 10th January. | 11th January. | 13th January. | 14th January. | 16th January. | 17th January. | 18th January. | 20th January. | 28th January. | 29th January. | 30th January. | 31st January. | 1st February. | 2d February. | 3d February. | 4th February. | 5th February. | 6th February. | 7th February. | 8th February. | 12th February. | 15th February. | 16th February. | 23d February. | 24th February. | 2d March. | 3d March. | 8th March. | 10th March. | 11th March. | 17th March. | 14th November. | Total of Men. | Women: 21st January. | Women: 5th February. | Women: 6th March. | General Total of Men and Women. |
|---|---|---|---|---|---|---|---|---|---|---|---|---|---|---|---|---|---|---|---|---|---|---|---|---|---|---|---|---|---|---|---|---|---|---|---|---|---|---|---|---|---|
| Number admitted each day. | 13 | 2 | 4 | 3 | 2 | 4 | 1 | 4 | 3 | 1 | 4 | 2 | 2 | 3 | 3 | 6 | 5 | 3 | 10 | 5 | 2 | 1 | 4 | 2 | 1 | 1 | 1 | 1 | 2 | 1 | 1 | 1 | 1 | 1 | 1 | 1 | 102 | 1 | 1 | 1 | 105 |

GENERAL ABSTRACT of Sick, from His Majesty's Ship PYRAMUS, treated in Genl Detacht Hospital, at ANTIGUA.

| | Officers, Seamen, and Marines. | | | | | | | | | | | | | | | | | | Women. | | | |
|---|---|---|---|---|---|---|---|---|---|---|---|---|---|---|---|---|---|---|---|---|---|---|
| | Febris Continua Com. | Febris Continua Icterades. | Phlegmon et Abscessus. | Enteritis. | Pneumonia. | Hepatitis Acut. | Podagra. | Rheumatism Acut. | Phthisis Pulmon. | Catarrhus Acut. | Dysenteria Acut. | Diarrhœa. | Melancholia. | Obstipatio. | Contusio. | General Debility. | Inebriari. | Total Men. | Febris Cont. Com. | Febris Cont. Icterades. | Total Women. | General Total. |
| Admitted between the 3d Jan. and 20th Dec. 1822........ | 11 | 102 | 1 | 4 | 1 | 2 | 1 | 2 | 2 | 4 | 1 | 14 | 1 | 1 | 1 | 4 | | 153 | 1 | 3 | 4 | 157 |
| Discharged between the 3d Jan. and 20th Dec. 1822...... | 11 | 72 | 1 | 4 | 1 | 2 | 1 | 2 | 1 | 4 | 1 | 14 | 1 | 1 | | 4 | 1 | 121 | 1 | 1 | 2 | 123 |
| Died between the 3d Jan. and 20th Dec. 1822............ | | 30 | | | | | | | 1 | | | | | | | | | 31 | | 2 | 2 | 33 |
| Remaining on the 20th December, 1822................ | | | | | | | | | | | | | | | 1 | | | 1 | | | | 1 |

ABSTRACT of Military Sick Recruits, daily admitted into General Detachment Hospital from the Transport in English Harbour, and the Encampment on Fort Berkly Isthmus, ANTIGUA.

| 1822. | From Transport. | | | From Encampment | | | | | |
|---|---|---|---|---|---|---|---|---|---|
| | 9th March. | 10th March. | 11th March. | 12th March. | 13th March. | 16th Marc h | 20th March | 23d March | Total Number. |
| Number admitted each day.... | 7 | 3 | 8 | 4 | 2 | 1 | 1 | 1 | 27 |

GENERAL ABSTRACT of Sick Recruits, from the Transport in English Harbour, and the Encampment on Fort Berkley Isthmus, ANTIGUA.

| | Officers & Soldiers. | | | | | |
|---|---|---|---|---|---|---|
| | Feb. Cont. Com. | Icterades. | Phlegmon et Abscess. | Rhumatis Acut. | Chron. | Total. |
| Admitted between the 9th & 23d March, 1822. | 2 | 27 | 1 | 1 | 1 | 32 |
| Discharged .............................. | 2 | 15 | 1 | 1 | 1 | 20 |
| Died .................................... | | 12 | | | | 12 |
| Remainiug................................ | | | | | | |

Signed—ROB. HARTLE,
Surgeon to the Forces,
Resident Medical Officer.

### APPENDIX C.

*Antigua,* 18*th September,* 1823.

My dear Doctor,

The circumstances respecting the sickness on board the Montagu Packet were these:—the Packet, on her passage home, in the latter end of December 1816, sprung so bad a leak that it was deemed proper to put back to St. Thomas's to have it stopped. During the process of heaving down, at a wharf on the other side of the harbour, opposite to the town, the packet sunk; the wharf was attached to a flat piece of land, backed by a small hill, covered by brushwood, and having some swampy ground at its base between it and the flat ground.

The passengers, ten in number, lodged in the town of St. Thomas until the packet was again ready for sea, which was in the space of three weeks. The crew lived in tents alongside the wharf, and underwent great fatigue in raising, heaving down, and refitting the vessel. At this period the ardent fever (commonly called yellow fever) prevailed to a considerable extent in the town of St. Thomas, but did not affect any of the passengers residing in town, while the officers and crew engaged on the opposite shore suffered by it; of the crew three died.

When the vessel was refitted, and even before she was thorougly dried, the passengers and sick, with one of the latter who had just died, were embarked. The packet immediately put to sea; had the fever been of a contagious nature there could scarcely have been a better opportunity for its exerting that influence; yet no case of fever took place during the passage home.

I remain, my dear Doctor,
Your's very truly,
ROBERT CRICHTON, M.D.

### APPENDIX D.

*Account of the Montserrat Epidemic of* 1821. By Richard H. Dyett, M.D.

Dear Musgrave, *Montserrat, September* 1*st,* 1823.

In furnishing you with a detailed account of our epidemic of 1821, I shall treat the different points connected with it agreeably to the order in which they are classed in your letter; subjoining such observations as the view which I entertain of the nature and importance of the subject will unavoidably elicit.

The 1st point embraces the origin of the epidemic, with the proofs of its non-importation.

The 2d, its symptoms and progress.

The 3d, its treatment.

The 4th, the classes of persons affected with the different degrees of concentration observed in each.

The epidemic was unquestionably not imported.

For several months previously to its appearance, the island was remarkably healthy. From the commencement of the year, until the melancholy period of its eruption, no death had been occasioned by fever, and after instituting the strictest inquiry, I was convinced, that no case of fever had been imported. Those who are acquainted with this colony—its inconsiderable size—its scanty population—the nature of my connexions—and the extent of my practice in it, will readily comprehend how easily and satisfactorily I could ascertain this fact; and how difficult, how nearly amounting to impossibility, it would be, to conceal from me the importation of a severe, or alarming malady.

To the epidemic of 1821, a local origin must therefore be assigned. Unhappily local causes, sufficient to account for its appearances existed at the moment. The weather was, and had been for many preceding weeks, intensely warm. The thermometer ranged in the shade from 89 to 96. No heavy rains had fallen, but the surface of the earth was daily moistened by light showers. In every corner of the town, but more particularly in the immediate vicinity of the house, in which the epidemic first manifested its presence, heaps of animal and vegetable filth commingled, were, to the disgrace of the police, suffered to accumulate. It is to the operation of these combined causes—to the production of a highly vitiated state of the atmosphere, arising from the action of extreme heat, upon mixed animal and vegetable matters, in a state of putrescency, and perhaps also to the extrication of miasmata, from the drying surface of the earth, that I attribute the generation of our late formidable and concentrated endemic.

The first case of fever occurred on the 5th May. The subject of it was a young European female, who had resided many years in the colony. It was ushered in by considerable heat and dryness of skin—loaded tongue—full rapid pulse—extreme restlessness—intense thirst—dull suffused watery eye—headache—uneasiness, with sense of stricture about the præcordia—and unconquerable irritability of stomach. On the third day the heat of skin disappeared—the pulse sunk to 80—the eyes, and subsequently the whole surface of the body became yellow. The disease terminated fatally on the 5th with black vomit—black stools—dry furred tongue—subsultus tendinum—hiccough—vibices, and intestinal hæmorrhage. A sister of this young lady was immediately afterwards attacked by the disease, which exhibited nearly the same symptoms, ran the same course, and produced the same fatal result. The rest of the white family consisting of eleven persons, most of whom were Europeans, fled in alarm to the country.

The epidemic which had thus commenced its ravages, now appeared in another, and distant quarter of the town, over which it continued for several months to extend, re-iterating its attacks, not rapidly, but gradually and generally, and in many instances destructively. During its course, 201 cases came under my care; among these there were fourteen deaths.

The violence of the epidemic had at one period considerably abated, and during the temporary calm, the family originally attacked, who had resided for upwards of six weeks at a distance in the country, ventured to return to the habitation, from which they had been so alarmingly expelled. Whilst absent from it, they had enjoyed complete, and universal immunity. In less than a week after they had retaken possession of their former dwelling, the disease renewed its visit, attacked four of their number, nearly at the same moment, and, in the person of the eldest sister, consigned another, and in this instance particularly, deeply lamented victim to an untimely grave. The others were more fortunate, they survived the attack; and the family, having again removed from the source of contagion, were exempt from further molestation, during the prevalence of the epidemic.

It could hardly be expected, that the attacks of a disease so general in its diffusion, and so persevering in its visitation, would, in a West Indian town, so ill-regulated as ours then was, be long limited to its range, or confined within its precincts. The intercourse with the country was as usual unrestricted. It never suffered even momentary interruption. The communication with the neighbouring islands, was not interdicted; nor was any restraint laid upon the crews of the European vessels in the harbour. Under these circumstances, it would have been almost miraculous, had the disease not spread into the country, or been introduced on board the ships. yet in the latter, only two cases of fever occurred, both of which were slight; and of the inhabitants of the country, none, but those who had incautiously exposed themselves to the nocturnal influence of the contagion, became its subjects. These cases were, with very few exceptions, confined to the military of the garrison stationed at a short distance from the town; and among them, the disease raged with extreme violence, and uncommon fatality.*

In the severe and fatal cases of this fever, the symptoms were, with some occasional difference, and modification, very generally the same, with those which I have described, as ushering in, and accompanying, the first case of the epidemic. In one only, could the irritability of stomach be conquered before death. Some died delirious—others comatose. Some, in the perfect possession of their senses, sunk quietly without struggle or convulsion; whilst, in one or two cases, the last scene was closed with strong convulsions. The appearances of herpes labialis was invariably followed by convalescence; but every case, in which an eruption resembling rupia simplex took place about the nose and mouth, terminated unfavourably. Most of the patients died on the 5th day. Some on the 7th, two on the 3d, and one on the 30th. This last was a singular case, the subject of it was a young Mongiel girl. The epidemic attacked her in all its malignity. The disease was characterised by extreme irritability of stomach—violent headach—constant delirium, with the "sputatio indecora in adstantes"—bloated countenance—low quick pulse—great restlessness—occasional sighing—black furred tongue horridly offensive breath—great debility, with tendency to syncope, on the slightest muscular exertion—hiccough—black stools—black vomit—hæmorrhagy from the nose, mouth and anus. The worst of these symptoms were present by the 9th day, yet she struggled through them all. On the 11th, her stomach became retentive, and she was evidently convalescent on the 14th; unfortunately, however, she was attacked by

---

* *Note by Dr. Musgrave.*—Its progress among the military exemplified in the strongest manner how completely local was the source of this disease, and how entirely incommunicable it was by personal contagion. I find on examining the reports of Dr. Hartle, and Hospital Assistant Parry, (the former of whom went to Montserrat, for the purpose of investigating the nature of the epidemic, and the latter was residing there in charge of the detachment) that from the 5th of May, to the 26th June, only two cases occurred among the troops, which were stationed at some distance from Plymouth on a healthy eminence; and these two there was every reason to suspect, from their intemperate charaeters, had eluded the vigilance of their officers and visited the town during the night. Unfortunately, however, Assistant Commissary General France, having in opposition to Mr. Parry's advice removed the public stores with his family from this healthy situation to the town, while the fever was raging there; first lost his daughter, and afterwards himself fell a victim to it on the 23d of June; "the yellowness (says Mr. Parry) appeared as early as the 2nd day; black matters were vomited on the 3d, and black stools were passed. On the 4th, the countenance became livid and bloated; there was lividness of the gums with hæmorrhage from the mouth and bowels; the disease terminated on the 5th, with marks of extreme putrescency."

Mr. France's fate proved a harbinger of evil to the detachment. On the 19th, in consequence of his illness, the officer in command felt it his duty to provide for the safety of the stores, &c. by ordering a corporal's guard to be placed over them, till they could be again conveyed to their former situation. Between the 19th and 24th of June, sixteen men were employed on this duty, each of whom passed a night in town. The consequence might with certainty have been predicted. From the 26th June, to the 4th July, a period of nine days, ten cases presented themselves under the most concentrated form, of which a lamentable proportion proved fatal; the scene being closed in every instance with the same marks of malignancy already enumerated as accompanying the dissolution of Mr. France. On the 24th of June, the guard was discontinued, and effectual measures were taken to prevent further intercourse with the town, especially during the night. Had personal contagion, however, contributed in the most trifling degree to its propagation, what could precautionary measures against intercourse with any place have availed, when the disease was *already among the men*, and the *communication between the infected and the sound continued unrestricted*? Yet the fact is indisputable, that in ten days after these measures were enforced, the progress of this dreadful fever entirely ceased, as the last case went into hospital on the 4th of July.

Dr. Hartle closes his report with these words, "nothing like the most distant shade of contagion could be traced in either the civilian or military. The steward of the hospital is a European, and was in constant attendance on the sick. Mr. Parry, exclusive of his kind and unremitted attention examined each case after death, The officers paid their customary visits to the hospital, yet the disease was not communicated to any."

Mr. Parry also thus expresses his sentiments. "No precautionary measures were adopted to interdict the general intercourse; no one apprehended, or even believed in the existence of contagion. In short, no circumstance occurred either among the inhabitants or troops that could afford the slightest ground for believing in the existence of such properties."

quotidian intermittent on the 20th, which, notwithstanding the liberal administration of bark, wine, and cordials, finally put a period to her existence—she died anasarcous. In the treatment of the epidemic, I relied principally upon blood-letting, calomel, and the cold bath. From the employment of the lancet, I was not deterred by considerations of age, sex, complexion, climate, or country. It was practised with the native, as well as the European; the Mulatto, as well as the white; the female, as well as the male; the child, as well as the adult. The objections to its use I considered unfounded. I regarded them as more theoretical than practical, and I was not deceived in my estimate of its general utility. It never appeared to do mischief; and whenever blood was drawn early and freely in the disease, the relief obtained by it, though sometimes only temporary, was always immediate, and manifest.

In the practice of this remedy, I was guided, and regulated by the severity of the symptoms, the suddenness of the attack, and the importance of the organ which it menaced; and I preferred bleeding the patient in an erect posture "ad deliquium." When thus practised, I have seen it suddenly arrest a violent paroxysm of fever, remove headache, and irritability of stomach, as if by enchantment, occasion immediate, general, equable, and continued perspiration, and by allowing an opportunity for the rapid, and liberal, administration of bark, restore the patient to health without the recurrence of fever, and without the production of that debility, which some medical writers have more fancifully, than truly, described, as consequent upon its employment in tropical climates.

After the first six and thirty hours however, the lancet was entirely laid aside, and the cure confided to other remedies. Among these, the cold affusion was one, from which the patient experienced the greatest benefit. I recurred to it in almost every case at the commencement of the disease, repeating it during the first 48 hours, as frequently as circumstances, or the situation of the patient would permit. When the affusion appeared to have been fruitlessly, or fatiguingly practised, a large and commodious tub, filled with cold water, was placed by the bedside of the patient; and directions were given, to immerse him in it for five minutes, as often as the heat, and dryness of skin, returned. As auxiliary to this practice, spunging with cold water and vinegar was recommended; only such clothing as decency required was allowed, and the bed was placed in the immediate neighbourhood of a window, which remained open both by day and night.

In conjunction with cold thus remedially directed, calomel in every severe case, and in every stage of the disease except the last, was largely exhibited. In some of those whose terminations were fortunate, from 300 to 500 grains were exhibited; and in no case did its conjunction with the cold treatment, appear either during the illness, or convalescence of the patient, to have been injurious. From 5 to 20 grains, according to the age of the patient, or the severity of the attack, were given in pill or powder, combined with pulvis aromaticus, or the aromatic electuary every two, three, or four hours, until the bowels were freely evacuated, and the system affected; or until perseverance in the remedy was evidently unavailing. This practice was eminently successful, although cases did occur, where even salivation, though early induced, could not avert the fatal termination of the malady.

In the slighter cases of fever, where irritability of stomach was either not present, or not distressing, other purgative medicines, such as jalap, infusion of senna, or cathartic extract, were beneficially employed; a solution of the disease being readily procured by their agency, in co-operation with the cold bath.

When the disease proceeded to its last dreadful stage, numerous and multifarious were the remedies to which I had recourse. A variety of medicines of the tonic, stimulant, and antiseptic classes was tried, and, I regret to say, but too frequently tried in vain. In the contest with the grim tyrant, Nature occasionally achieved an unexpected victory, but art had, I am willing to acknowledge, no triumphs to record. I can mention no remedy which seemed to possess the slightest control over the disease, after the occurrence of black vomit. In two desperate cases indeed, when yellowness of the skin had early appeared, and the supervention of the horrid symptom above-mentioned was with reason apprehended, the exhibition of a large dose of calcined magnesia in mint tea, with the addition of a drachm of the spiritus laven. dulc. compositus (which remained upon the stomach, and produced severe bilious diarrhœa) seemed conducive to the recovery of the patient.

All classes in the community, both male and female, were invaded by the epidemic, but with different degrees of severity, and varieties of concentration. Of the 201 cases which I attended, 42 were whites—110 people of colour—49 blacks. Among these latter few of the cases were severe, and all of them ended favourably. Some of the most violent, and best marked cases of yellow fever, occurred among the people of colour; four of whom became its victims, and died with black vomit, and other malignant symptoms. Of the 42 whites who were attacked, four were Europeans lately arrived, and all perished. Twelve were Europeans several years resident in the island; of these three died. Twenty-six were natives, of whom 23 recovered. Of the aggregate of those who died, six were males, six females, and two were children under four years of age.

In the greater number of cases, the type of fever was decidedly remittent; but the symptoms were as malignant, and the terminations as fatal in these, as in the cases of continued fever. In one instance the disease made its appearance under the form of regular intermittent. It commenced with rigors, succeeded by heat, and the paroxysm terminated in eight hours by profuse perspiration. On the following day the intermittent paroxysm returned with a similar solution. On the third day the patient complained of extreme debility, and her eyes and skin became yellow. She died on the fifth day with black vomit and hæmorrhagy from the nose and mouth. In the case of one of my own children, a girl about six years of age, the disease commenced with excruciating headache, flushed countenance, distressing restlessness, incessant retching and vomiting. The frontal branch of the temporal artery was immediataly opened, and six ounces of blood taken from it. The headache, flushed countenance, and restlessness, soon disappeared. The stomach became retentive, and although the deepest jaundice I ever saw supervened on the seventh day, the disease, under the type of infantile remittent, ran its course safely in fourteen days.

No infectious property was attached to this epidemic. It spread solely by atmospheric contagion. During its prevalence, so numerous, positive, and unequivocal were the proofs afforded of this truth, that into the minds even of the most timid no dread of personal infection ever intruded. The most anxious and solicitous attention was therefore universally and beneficially bestowed upon those who were unfortunately subjected to its influence. I have to record, and record with pleasure, that none of the persons attacked were attendants upon the sick. Those whose duty or inclination led them to bestow their care and attention upon the sufferers, uniformly escaped. Many of these attendants were persons highly predisposed to febrile disease. To enumerate all the cases of this kind would be tedious and unnecessary. "Ab paucis disce omnes."

At the commencement of the epidemic, a fine European boy, who had resided but a very few months in the colony, was attacked by the disease in all its violence and malignity; and immediately removed into the country. His sister, who had also but lately arrived, was naturally desirous to attend her brother. I was asked whether she might do it with safety, and my reply was unhesitatingly given in the affirmative. During the four days of his illness, she attended him with the most unremitting and affectionate solicitude. His cheek often rested upon her's, and his head was often pillowed upon her bosom. He expired in her arms the fifth day. Another European lady shared in the sister's devoted attention to this boy, yet the disease was not communicated to either.

In August 1821, an European lad of the name of Farrile went on board the Arethusa, Captain Norie, on his passage to England. The day after the sailing of the vessel he sickened, became yellow on the third, and died with black vomit on the seventh day. No other individual on board was attacked.

Towards the decline of the epidemic, several ladies and gentlemen, whilst riding out on a party of pleasure, were surprised by a shower of rain. Some of the party had lately arrived from Europe. Two of the ladies, both natives of the island, and long resident in it, refused for some hours to change their clothes. They, and only they, were in a few days after attacked with fever, and both died. One of these ladies I visited on the third day of her illness, and attended until her dissolution; and I think I never saw a better or stronger marked case of malignant yellow fever. Yet, although the family, of which both the ladies formed part, was a numerous one, the disease was not communiated to any of the other members.*

In addition to the proofs afforded by the epidemic of 1821, that the concentrated endemic of this country is not infectious, I shall briefly advert, in confirmation of the fact, to the evidence which the ten following cases supply. In July 1811, John Bankes, a young stout black man, was, after several hours of continued exposure to a very hot sun, seized with malignant fever, and died on the fourteenth day with black vomit, black stools, and intestinal hæmorrhage. This man, who was confined in a small, ill-ventilated apartment, was so numerously attended, that I was repeatedly obliged to order the greater number of his nurses out of the room. None of his attendants suffered, nor was there, during the course of the year, another case of the concentrated endemic. In October 1812, I was called to visit a sailor labouring under yellow fever, by which he had been attacked two days before, on his passage to this island from St. Eustatius. This man was perfectly yellow when I first saw him. His skin was soft and cool. His pulse between 70 and 80: but he could retain upon his stomach neither food nor medicine; and died with the most malignant symptoms, on the fifth day after his arrival. The disease died with him.

Will my reasoning also be deemed shallow and perverted? Shall I be accused of originating baneful impressions from the symptoms which I have described? From the circumstances which I have related, and the facts which I have detailed, I do not hesitate to deduce the following inferences:—

1st. That the epidemic of 1821, in this island, was unquestionably not an imported disease.

---

* In examining closely the gums of one of my patients, in the last stage of fever, to see whether they had been affected by the calomel, she sighed heavily. Her breath was horridly offensive, and the air penetrated into my nostrils. I do not remember ever having received a severer nervous shock. So great also was the irritation occasioned in the throat and fauces, that it was upwards of two hours and a half before I ceased coughing and spitting.

2d. That it derived its origin from local contagion, and assumed the form of decided yellow fever.

3d. That there exists no difference whatever in kind, but merely a difference in degree, between yellow fever and the common remittent of the climate.

4th. That this remittent, bilious remittent, concentrated endemic, yellow, American, Cadiz, Gibraltar, Grenada, or Bulam fever, "aut quocunque alio nomine gaudet," is not infectious—not personally communicable, but propagated by atmospheric contagion *solely*.

R. W. DYETT, M. D.

### APPENDIX E.

*Extract from a Communication addressed by His Excellency Sir F. P. Robinson, the Governor, to the Honorable the Speaker of the Assembly of the Island of Tobago, dated January 6th,* 1821.

Sir,

The following subjects I consider it my duty to submit, through you, to the House of General Assembly, in the confident hope that they will be considered in the light they merit, and that I shall be enabled, through the wisdom of the Legislature, to effect the desirable purpose of relieving the colony, as far as human means can do, from the dreadful scourge with which it has been afflicted for these last two years. *Bacolet Swamp has long been considered as the great source of sickness both in the town and garrison.* If I had entertained any doubts as to the certainty of it, they would have been removed by the survey and experiments lately made by Captain Smith, of the Royal Engineers, *by which the cause of the fever has been placed in such a decided point of view*, that any further delay in endeavouring to effect its removal, would be altogether unpardonable.

"What is called poor land, lying between the cultivated ground of Bacolet and Friends Field Estate, and the King's Ground, requires to be kept constantly clear of brush and long grass; because the noxious vapour from the swamp lodges in those places, in the first instance, and by the subsequent operation of the heat of the sun, it *floats through the air, and occasions a mortality that appals the stoutest hearts.*"

(Signed) F. P. ROBINSON, *Governor.*

*Extracts from a Letter addressed by Doctor James Anderson, of Trinidad, to Lieutenant-Colonel Young, commandimg the Garrison there, dated November 9th,* 1820.

Sir,

As I understand, by late accounts from Tobago, that great sickness and mortality have prevailed in that island for many months past, and that they still continue their ravages in the form of fever, attended with the most fatal consequences; I beg leave to state to you, as commanding His Majesty's garrison in this island, and request the favor of you to communicate it, for the information of His Excellency Sir Frederick Robinson, Commander in Chief on the Station, what I conceive may be of great service in putting a stop to, or preventing, in future, this dreadful scourge to our troops stationed on Fort King George.

In the year 1796, when surgeon of the 4th battalion, 60th regiment, I was for some time stationed in that island, and had an opportunity of observing the fever prevailing in Fort King George, which proved fatal to the Major of the Regiment, and many men; and several of the officers had most violent attacks, all of the same species of fever, but recovered. I had no doubt, in my own mind, as to the cause of the disease, and in ascribing it to the marshy grounds situated to the windward of Fort King George, and when I next year quitted the regiment on account of ill health, and was placed on half pay, I published at Edinburgh some Observations on Yellow Fever of the West Indies, in which I had occasion to take notice of the particular situation of the fort in Tobago. I shall beg leave to give you an extract from that pamphlet, of what relates to our present subject. "Fort King George, in which we were stationed, is situated on a high hill, commanding the town and harbour of Scarborough, in a bay to leeward of it. To windward of the fort, there are considerable tracts of low swampy ground, and as we know from many facts that marsh miasmata can be carried to a very considerable distance and even to remarkably high situations, we can easily suppose the marsh miasmata to be translated by the constant trade wind along the gradual ascent from those swamps to Fort King George, and there occasion the frequency of this fever on that situation. This is further confirmed by that part of the town which lies at the foot of the hill immediately under the fort, being much more healthy than the fort itself, although the air is much hotter in the town, and it is in a great measure deprived of the refreshing breezes which keep the air at the fort moderately cool—which circumstance I would thus account for, that the miasmata were carried over by the current of wind considerably above and beyond the town, and of course could not affect it; although great numbers were attacked with this fever, yet I never could perceive any marks of the disease being propagated by contagion, and I could only impute its prevalence to the men being exposed to the action of the marsh miasmata."

(Signed) JAMES ANDERSON.
Surgeon H.P. 60th Foot.

*Extract from a Letter, addressed by Captain W. D. Smith, commanding Royal Engineers, to His Excellency Sir F. P. Robinson, dated Tobago, 12th January,* 1821.

Sir,

In compliance with the commands of your Excellency, I have the honor to enclose a report on the swampy ground on the Estate Bacolet.

Sensible of the vast importance of the object which your Excellency holds in view, no *less than that of obviating a recurrence of that inveterate disease,* which has recently proved so fatal to many brave officers and men, who composed the army under the command of your Excellency; I have paid all possible attention in framing my report, &c.

(Signed) W. D. SMITH.
*Commanding R. Engineers.*

*Extract from Dr. Hartle's Report for June,* 1821.

It would perhaps be worthy of remark, that His Excellency Major General Sir F. P. Robinson's family, residing at Government House, Tobago, consisted of His Excellency, Lady Robinson, one son, four daughters, and a European lady; out of these eight persons, only four were attacked with this (said to be) contagious fever, two of whom died.

Those attacked were His Excellency, son and two daughters. It is but natural to conclude, that her ladyship and daughters could not be deficient in attention to so material a proportion of their family; yet her ladyship, two daughters, and the young European lady escaped the disease altogether. Another strong proof is, that of the Fort Adjutant Lieut. Buchanan, who, with his wife and sister resided in Fort King George; his sister was attacked with the prevailing fever, of which she died. Lieut. and Mrs. Buchanan constantly, and with every marked affection, attended her during her illness, yet neither of them were attacked with the fever. Major Watts, Deputy Assistant Quarter and Barrack Master General, was ordered from this island to Tobago, on duty of his department; he resided with Lieut. Buchanan, in Fort King George, and was taken ill. Lieut. Buchanan was ordered to quit the quarter, which he did do, but went repeatedly to visit, and take care of his friend Major Watts, yet this second time of being exposed to the fever had no avail, and he again escaped.

The hospital steward, who was a European corporal, and must have been constantly exposed to the said animal contagion, lost a child with this fever, yet he was not attacked.

*Another Extract from the same Report.*

"The Inspector of Hospitals, Mr. Green, having favoured me with the sight of a nominal list of those who suffered with the fever at Barbadoes, I remarked, that the greatest proportion of them were persons who *resided in the huts and that neighbourhood.* Those huts, and that lower part of the garrison to my knowledge have more than once been the cause of great sickness and mortality at St. Ann's. They cannot be otherwise, for they *stand on the borders of a swamp or bog of no small extent,* and the dwellings called the huts were always miserably bad. In my opinion, as long as they are allowed to be inhabited, they will be the cause of great sickness and mortality. I understand it has been said, that the fever was imported to Barbadoes by a man from Tobago, but I should be much more inclined to believe, that it was reared in the superficially dry swampy ground, and nourished in the huts."

---

### II.

### *An Account of the Effect of Mercurial Vapours on the Crew of His Majesty's Ship Triumph, in the year* 1810, *by* William Burnett, M.D. *late Physician and Inspector of Hospitals to the Mediterranean Fleet, and now one of the Medical Commissioners of the Navy.*

[Communicated to the Royal Society by Matthew Baillie, M.D. F.R.S.]

It has long been known, that in the vacuum of the barometer, mercury rises in a vaporous state, at the usual temperature of this climate, and that persons employed in the mines from whence this metal is procured, as well as those who are employed in gilding and plating, have suffered paralytic, and other constitutional affections, from inhaling the air saturated with mercurial vapours; had any doubt remained of mercury existing in the state alluded to, it would be effectually removed by the experiments made by Mr. Farraday, detailed in the 20th number of the Journal of Science, &c.*

An unprecedented event which occurred in one of His Majesty's ships of the line at Cadiz, in the year 1810, a short time before I took upon me the charge of the medical department of the Mediterranean fleet, has afforded me an opportunity of illustrating this subject on a very extensive scale, the details of which may not perhaps be uninteresting to the Royal Society.

The Triumph, of 74 guns, arrived in the harbour of Cadiz, in the month of February, 1810; and in the following March, a Spanish vessel laden with quicksilver, for the mines in South America, having been driven on shore in a gale of wind, and wrecked under the batteries then in possession of the French, the boats of this ship were sent to her assistance, by which means, during many successive nights, about 130 tons of the quicksilver were saved, and carried on board the Triumph, where the boxes containing it were principally stowed in the bread room.

The mercury, it appears, was first confined in bladders, the bladders in small barrels, and the barrels in boxes. The heat of the weather was at this time considerable, and the bladders having been wetted in their removal from the wreck soon rotted, and the mercury, to the amount of several tons, was speedily diffused through the ship, mixing with the bread, and more or less with the other provisions. The effect of this accident was soon seen by a great number of the ship's crew, as well as several of the officers being severely affected with ptyalism, the surgeon and purser being amongst the first, and most severely affected, by the mercury's flowing constantly into their cabins from the bread room, their cabins being, as is usual, on the orlop deck, separated from this store by partitions of wood. In the space of three weeks, from the mercury's being received on board, two hundred men were afflicted with ptyalism, ulcerations of the mouth, partial paralysis in many instances, and bowel complaints.—These men were removed into transports, where those more slightly affected, soon got well; but fresh cases occurring daily, Rear Admiral Pickmore, then in command of the squadron, ordered an inspection to be made by the surgeons thereof, and in consequence of their report, sent the Triumph to Gibraltar, to remove the provisions, and purify the ship by ablution, the affected men being sent to the naval hospital, which order was strictly attended to, the provisions, stores, and likewise the shingle ballast, being removed on shore.

Notwithstanding the removal of the provisions, &c. and afterwards frequent ablution, on re-stowing the hold, every man so employed, as well as those in the steward's room, were attacked with ptyalism; and during the ship's passage, and on her return to Cadiz, the fresh attacks were daily and numerous, till the 13th of June, when the Triumph sailed for England. After their departure from Cadiz, they experienced fresh breezes from the N.E. and the men being kept constantly on deck, the ship aired night and day by wind-sails; the lower deck ports allowed to remain open at all times, when it could be done with safety: allowing no one to sleep on the orlop deck, and none affected with ptyalism on the lower deck, a very sensible decrease in the numbers daily attacked soon became apparent; but nevertheless, many of those already affected became worse, and they were under the necessity of removing twenty seamen, and the same number of marines, with two serjeants and two corporals to a sloop of war, and the transports in company. On their arrival in Cawsand Bay, near Plymouth, on the 5th July, not one remained on the list for ptyalism. The effects of the mercurial atmosphere was not confined to the officers and ship's company; almost all the stock consisting of sheep, pigs, goats and poultry died from it; mice, cats, a dog, and even a canary bird shared the same fate, though the food of the latter was kept in a bottle closely corked up.

The surgeon (Mr. Plowman) informed me in conversation, that he had seen mice come into the ward room, leap up to some height, and fall dead on the deck.

The Triumph, previous to this event, had suffered considerably by having a number of her men attacked with malignant ulcer, which at one time prevailed to a considerable extent in our ships both at home and abroad, and in many of the men who had so suffered, the ulcers which had long been completely healed, without even an erasure of the skin, broke out again, and soon put on a gangrenous appearance.

The vapour was very deleterious to those having any tendency to pulmonic affections: three men died of phthisis pulmonalis, who had never complained, or been in the list before they were saturated with the mercury, and one man who had suffered from pneumonia, but was perfectly cured, and another who had not had any pulmonic complaint before, were left behind at Gibraltar, labouring under confirmed phthisis. Two only out of so large a number affected, died from ptyalism, gangrene having taken place in their cheeks and tongues; they had previously lost all their teeth. In the case of a woman who was confined to bed in the cockpit, with a fractured limb, not only were all the teeth lost, but many exfoliations also took place from the upper and lower jaws.

The mercury shewed its effects on the ship herself, by the decks being covered with a black powder, but quicksilver was not discovered at any time in this powder in a native or globular state, though the brass cocks of the boilers, and the copper bolts of the ship, were covered with the metal, the last to some extent within the wood. A gold watch, gold and silver money kept in a drawer, and likewise some of the iron-work of the ship which had been kept bright, evidently shewed the influence of the prevailing atmosphere, being in some places covered with quicksilver.

In a communication, with which Mr. Plowman, the surgeon of the Triumph has obliged me, he states, that those who messed and slept on the orlop and lower decks, with the exception of the midshipmen, suffered equally, while those on the main or upper deck were not so severely affected; the men who lived and slept under the fore-castle escaped with a slight affection of the gums. The only reasons which can be assigned for the partial escape of the midshipmen are, that the wind-sails were kept always in action, and these gentlemen were almost constantly on deck, or were more frequently employed on service out of the ship, in proportion to their numbers, than the men.

Various opinions were entertained of the manner in which the systems of the sufferers were brought under the influence of the mercury. By some, it was supposed to have originated from the use of the bread and other provisions, with which the mercury had mixed itself, and to such an extent was this opinion carried, that I find, by reference to official documents in the Victualling Office, that seven thousand nine hundred and forty pounds of biscuit were condemned as unserviceable, from having *quicksilver mixed with it.*

By others, amongst whom was Mr. Plowman, the surgeon, it was considered to have arisen from inhaling the mercurialized atmosphere; and, from the preceding details, I think there cannot remain a doubt, that this opinion was the true one.

It is well known, that mercury in its native state has often been administered in very large doses, in cases of obstinate constipation, without producing any specific effect on the system, merely removing the affection by its specific gravity. I have, however, reason to believe, from the accounts of Orfila and others, that if the mercury was to be retained in the intestines for some time, and thus subjected to the action of the contents of the stomach and bowels, a part might become oxydated, and being conveyed into the system by means of the absorbents, would there shew its specific effects. But after the removal of the provisions, &c. at Gibraltar, many fresh cases occurred, and many relapses amongst those who had been cured out of the ship took place, on their return to duty on board, which effectually destroys the probability of this having been the cause of the succeeding ptyalism and other morbid affections.

It only remains for me to offer my opinion of the manner in which the system became saturated by the mercury, and this I conceive to have been effected by inhaling the mercurial vapours, the quicksilver being then in the most perfect state of division, was readily taken up by the absorbents of the lungs, and soon shewed its influence on the system generally. This idea is very much strengthened by the effect which was produced on the animals on board already mentioned, as well as by the circumstance of a great number of men being attacked after the ship was cleared at Gibraltar, and till she arrived in a more northern latitude.

It may be considered out of place here, to give any detail of the curative means employed; I shall therefore only briefly state, that sulphur, given in large quantities internally, produced no alleviation of the symptoms; on the contrary, it greatly augmented the bowel complaints, with which many of the men were affected, and brought on a most severe tenesmus; consequently it was laid aside; applied externally, it was of no use.

The only plan which produced effectual relief, was removal from the ship, with the frequent use of small doses of neutral salts and detergent gargles.

W. BURNETT.

